# A High Sensitivity FBG Strain Sensor Based on Flexible Hinge

**DOI:** 10.3390/s19081931

**Published:** 2019-04-24

**Authors:** Mingyao Liu, Wenzhi Wang, Han Song, Shiguang Zhou, Weijian Zhou

**Affiliations:** 1 School of Mechanical and Electrical Engineering, Wuhan University of Technology, Wuhan 430070, Hubei, China; lmylyf@126.com (M.L.); wangwz99@163.com (W.W.); sgzhou@whut.edu.cn (S.Z.); rodgeratzwj@whut.edu.cn (W.Z.); 2 Hubei Digital Manufacturing Key Laboratory, Wuhan University of Technology, Wuhan 430070, Hubei, China

**Keywords:** FBG-based strain sensor, flexible hinge, sensitization, flexibility matrix method

## Abstract

For the purpose of improving the sensitivity of the fiber Bragg grating (FBG)-based strain sensor. A novel FBG-based strain sensor with high sensibility was designed by means of a flexible hinge bridge displacement magnification structure. This sensor can be used to accurately measure the strain of a mechanical structure surface. In this paper, the strain sensitization amplification factor of the sensor was calculated by using the flexible matrix method and the strain energy theory. The magnification had been verified by using simulation analysis and experimental results, and the error between theoretical calculation and simulation analysis was less than 7%. The result shows that the strain sensitivity of the sensor is 10.84 pm/με, which is about 10 times to that of the bare FBG sensor. This sensor is sensitive to micro-strain, so it can be well applied to health monitoring of a mechanical system.

## 1. Introduction

Nowadays, machinery and equipment are developing in the direction of large-scale, integration, high-speed and automation. How to ensure the safe and reliable operation of the mechanical equipment, especially key and critical equipment, is directly related to the development of machinery manufacturing and economy. Therefore, it is of great practical significance to further study the scientific and technical problems of on-line monitoring and fault diagnosis for mechanical equipment, and further improve the level of scientific and technological monitoring for the condition monitoring and fault diagnosis of mechanical equipment [1,2].

The sensor system is a very important part of condition monitoring and fault diagnosis. In recent years, the optical fiber sensing technology has developed rapidly, which provides a new principle and method for the condition monitoring and fault diagnosis of the mechanical system. Fiber grating sensor has many advantages, such as small size, explosion-proof features, electrical insulation and anti-electromagnetic interference capability, high precision, high reliability, and environmental adaptability [3]. It is also possible to allow the real-time measurement of the operating status of “multi-point” mechanical system by arranging a plurality of sensors for different parameters on a single optical fiber to form a distributed sensing [4,5].

Many scholars around the world have studied the application of Fiber Bragg Grating (FBG) sensors. K. Kesavan et al. studies the apparent strain using FBG strain sensors for different structural materials [6]. In order to protect the delicate and easy-to-break sensing optical fiber and effectively sense the external strain, it is generally necessary to package the FBG. Zhao et al. took the case of an airship, as an example to analyze the strain transfer of surface-attached fiber Bragg grating sensors [7]. Hyung-Joon et al. used arrayed FBG sensors to measure wind turbine tower strain and bending deformation. Ten FBG sensors are arranged and installed on the inner surface of the tower in the main wind direction and the DST (displacement-strain transformation) matrix was used to convert the deflection of the tower top position [8]. After a lot of research, FBG is widely used in civil engineering [9], aerospace [10], petrochemical [11], large-scale port [12] and other fields [13]. In the field of mechanical systems, Zhou et al. introduced the concept of dynamic monitoring and fault diagnosis of fiber grating distributed in mechanical system, and put forward many kinds of applications of fiber grating in mechanical system [14]. However, due to its low sensitivity and easy breaking, the FBG sensors are still used in large civil engineering works and not the best choice for high precision measurement of a mechanical system.

In view of the lack of sensitivity in FBG detection, many researchers have carried out research. The sensitivity improvement of the FBG sensor mainly depends on special packaging. By using a reasonable packaging method, the physical quantity that is difficult to measure is converted into a physical quantity with high sensitivity, which can effectively improve the detection accuracy of the sensor [15]. Vengal Rao Pachava et al. used a metal diaphragm to convert the pressure measurement into a tensile force to the FBG, resulting in a pressure sensor with high sensitivity [16]. In addition, the use of polymer packaging can also improve grating strain sensitivity. Wen Qingzhen et al. used a polymer package for fiber grating to produce a fiber grating pressure sensor with high compressive strain sensitivity. Sensitivity of this sensor can reach −1.1 × 10^−3^ MPa^−1^ [17]. The tubular package with two-point clamping not only provides effective protection for the fiber grating but also provides a certain degree of sensitization to the sensor. Li et al. design a strain sensor with a tube package, with a sensitivity of 2.52 pm/με. 24 strain sensors and 6 temperature sensors were placed on the box girder of the expressway bridge for structural health monitoring for up to 5 months [18]. Gao et al. incorporate a flexible hinge in the package structure. The flexible hinges reasonably bear the overall deformation. The designed sensor strain sensitivity is 3.36 pm/με [19]. Some conventional linkages also have a displacement amplification function, and the substrate package based on the principle of the linkage mechanism can also improve the sensitivity of the strain sensor. R. Li et al. used a link-type lever amplifying structure to design a strain-sensitized sensor with a sensitivity of 6.12 pm/με [20]. Later, R. Li et al. improved the structure and designed a sensor with higher strain sensitivity. The sensor can be used not only for the measurement of tensile strain but also for compressive strain. The sensors have strain sensitivities of 7.25 pm/με and −2.94 pm/με, respectively [21]. J. Peng et al. also use lever mechanisms and flexible hinges to enhance sensor sensitivity. The continuous lever mechanism effectively amplifies the input displacement. The strain sensitivity of the sensor is 11.49 pm/με by calibration experiment [22].

However, the above sensors are either insufficiently sensitive or have a large structural size. In this paper, a new type of sensor is proposed, which not only has very high sensitivity but is also small in size, so it is well suited for use in conventional mechanical systems. In this sensor, the flexible hinge bridge displacement amplification structure is used to enhance the sensitivity of fiber grating strain measurement. Compared with the traditional FBG sensor, the sensor proposed in this paper is smaller in size, easier to install and has a very high strain magnification. It can be used for high precision measurement of mechanical systems. This article mainly introduces it from the following parts: the principle and mathematical model of sensor, the simulation analysis of sensor sensing characteristics and the experimental analysis.

## 2. Principle of the Sensors and Mathematical Model

### 2.1. Principle of FBG Sensing

Fiber Bragg gratings (FBG) are passive devices based in the modulation of the refractive index, along the optical fiber core.

The reflection wavelength *λ_B_* of a fiber Bragg grating is determined by the effective refractive index *N_eff_* of the fiber and the periodicity ***Λ*** of the fiber grating, which is given by the first order Bragg condition:

(1) $$\lambda_{B}=2N_{eff}\cdot \Lambda$$

where *N_eff_* is the optical fiber effective refractive index and ***Λ*** is the periodicity of the FBG.

The Bragg wavelength dependency on the temperature and strain could be described as follows, the first equation below represents the strain effect on the Bragg wavelength, expressed by Equation (2). The second equation below represents the Bragg dependency to the temperature, which can be expressed by Equation (3):

(2) $$\frac{\Delta \lambda_{B}}{\lambda_{B}}=\left(1-P_{e}\right)\cdot \Delta \varepsilon$$

(3) $$\frac{\Delta \lambda_{B}}{\lambda_{B}}=\left(\alpha +\xi \right)\cdot \Delta T$$

where Δ*ε* is the axial strain acting on the FBG, *P_e_* is the effective spring constant, and generally takes a constant of 0.22, Δ*T* is the change in ambient temperature, α is the thermal coefficient of the fiber, and *ξ* is the coefficient of thermal expansion of the fiber. For the FBG with a center wavelength of 1550 nm, the strain sensitivity is 1.2 pm/με, and the temperature sensitivity is 13 pm/°C.

Assuming that the grating wavelength changes caused by strain and temperature are independent of each other, when strain and temperature change at the same time, the relative change in the Bragg wavelength is expressed as:

(4) $$\frac{\Delta \lambda_{B}}{\lambda_{B}}=\left(1-P_{e}\right)\cdot \Delta \varepsilon +\left(\alpha +\xi \right)\cdot \Delta T$$

The wavelength shifts caused by the strain and temperature can’t be identified, when these two variables change at the same time. When measuring one of the physical quantities, it must be interfered by another physical quantity. For pure strain measurement, the change of temperature on the Bragg wavelength needs to be suitably compensated.

### 2.2. Measurement Principle of FBG Strain Sensor

**Figure 1:**
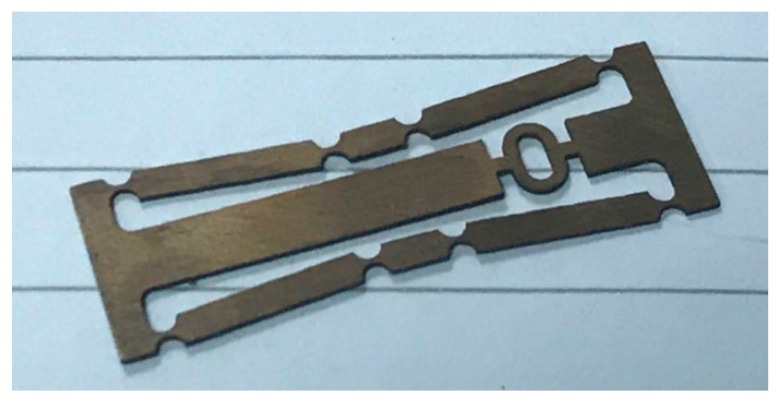
Real image of the designed sensor.

**Figure 2:**
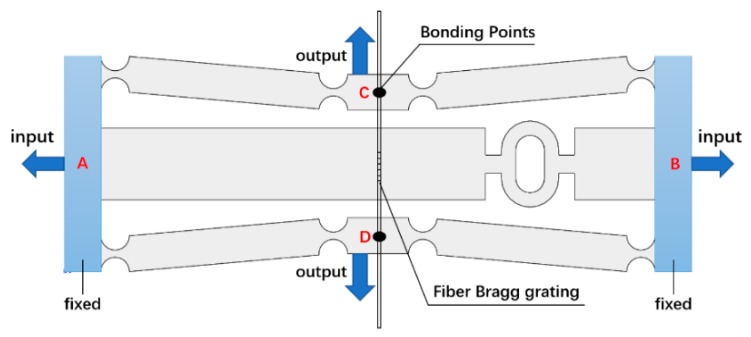
Schematic diagram of the sensor.

The manufactured specimen of the developed FBG strain sensor is shown in Figure 1 and the structural model of the FBG strain sensor is shown in Figure 2. During measurement, the fixed blocks (points A, B) at the left and right ends of the sensor are attached to the surface of the object to be tested. The fixed block moves with the object to feel the strain on the input direction in Figure 2. At the same time, the fiber grating is pasted on the structure in the middle by two-point bonding to measure the strain in the output direction of the sensitized substrate.

The sensitization capability of this FBG strain sensor consists of two parts. On the one hand, a bridge displacement amplifying mechanism is employed in the sensitizing substrate. The horizontal displacement **Δ*L_AB_*** that the sensor obtained will be amplified and converted into a displacement **Δ*L_CD_*** in the vertical direction. On the other hand, the use of the sensor can effectively shorten the sticking distance of the fiber grating. The shortened multiple is the ratio of the distance ***L_AB_*** between two points of AB to the distance ***L_CD_*** between two points of CD. The strain measured by the two-point bonded FBG is:

(5) $$\varepsilon^{*}=\frac{\Delta L_{CD}}{L_{CD}}=\frac{\Delta L_{CD}}{\Delta L_{AB}}\cdot \frac{L_{AB}}{L_{CD}}\cdot \varepsilon =k_{1}\cdot k_{2}\cdot \varepsilon$$

where *k*_1_ = Δ*L_CD_*/Δ*L_AB_* is the displacement magnification ratio and *k*_2_ = Δ*L_AB_*/Δ*L_CD_* is the paste shorten multiple. Under the influence of these two aspects, a high multiple sensitivity can be obtained.

### 2.3. Mathematical Model of the Sensor

The Castigliano’s second displacement theorem is used to calculate the structural model. Firstly, the relationship between the force and the deformation of a single flexure hinge is obtained. Secondly, the overall strain energy of the structure is calculated. Finally, the relationship between the force and the deformation at the end of the structure is obtained by introducing the second displacement theorem.

**Figure 3:**
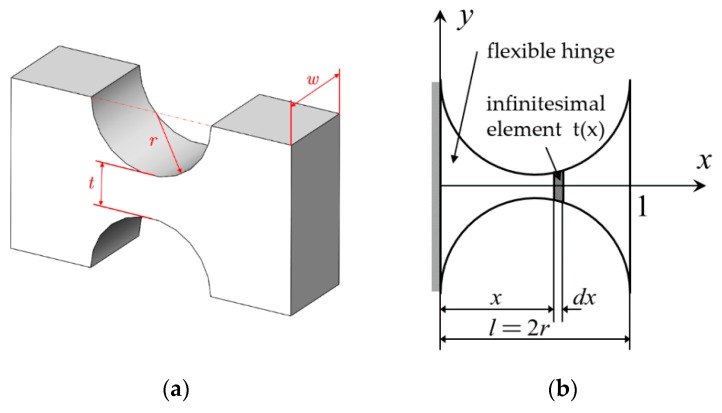
Flat single-axis straight circular flexible hinge. (**a**) Three-dimensional model, (**b**) Plane model.

Before the calculation of the whole structure, the force deformation relationship of a single flexible hinge is analyzed. Taking a straight circular flexible hinge as an example, the main dimensional parameters are the hinge radius *r* (half of the total length *l*), the narrowest width *t*, and the thickness *w*, as shown in Figure 3a. Generally, the overall thickness of the hinge is the same, so it is often projected into a two-dimensional plane, and analyzed by the micro-element method, as shown in Figure 3b. Only the width *t(x)* of the hinge varies with the change of *x*.

For a flat single-axis straight circular flexible hinge, the hinge width *t*(*x*), a cross-sectional area *A*(*x*), and moment of inertia *I*(*x*) can be expressed as:

(6) $$\{\begin{array}{l} t\left(x\right)=t+2\left(r-\sqrt{2rx-x^{2}}\right)\\ A\left(x\right)=w\cdot t\left(x\right)\\ I\left(x\right)=w\cdot t{\left(x\right)}^{3}/12 \end{array}$$

where ***t*** is the narrowest width of the hinge, and ***r*** is the radius of the straight circular hinge. The relationship between the deformation [***u***] and the force [***F***] at the point 1 of the hinge end in Figure 2b can be expressed as:

(7) $$\left[u\right]=C\cdot \left[F\right]=\left[\begin{array}{ccc} C_{11} & 0 & 0\\ 0 & C_{22} & C_{23}\\ 0 & C_{23} & C_{33} \end{array}\right]\cdot \left[F\right]$$

The flexibility matrix ***C*** of a flat single-axis straight circular flexible hinge has been given by Lobontiu [23]. Among them, **C**_11_, **C**_22_, **C**_23_ and **C**_33_ are mainly affected by the hinge thickness ***w***, the elastic modulus ***E***, and the hinge width ***t***(***x***), and given in Appendix A (A1).

**Figure 4:**
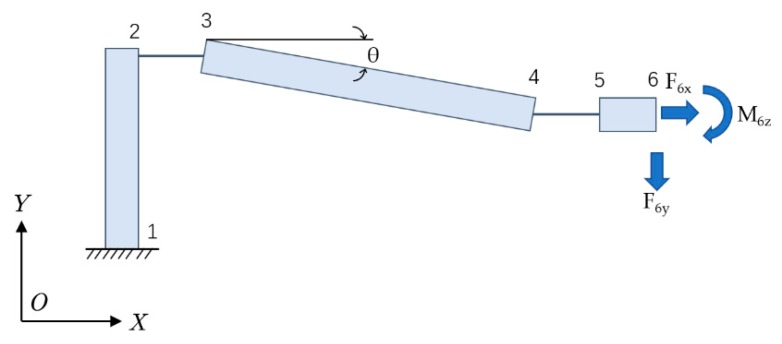
Bounding conditions and loading for quarter-model.

Due to the central symmetrical structure of the sensor, the calculation of the displacement amplification ratio is exemplified by a quarter structure. As shown in Figure 4, the 1/4 structure consists of five sections, including three rigid rods (part L_12_, L_34_ and L_56_) and two flexible hinges (part L_23_ and L_45_). The rigid rods won’t be out of shape and the total deformation of the structure is completely borne by the two flexible hinges. Assume that the left end point 1 of the 1/4 structure is fixed and the right end point 6 is the free. [F_6x_, F_6y_, M_6z_] are the load acting on the free end, [u_6x_, u_6y_, θ_6z_] are the deformation at the free end of the structure.

The total strain energy of the structure consists of three parts, which are respectively caused by axial load, shear and bending. It can be expressed as:

(8) $$U=U_{a}+U_{s}+U_{b}=\overset{n}{\underset{i=1}{\sum }}\left[\int_{0}^{l_{i}}\left(\frac{N_{i}{}^{2}}{2E_{i}A\left(x_{i}\right)}+\frac{\alpha S_{i}{}^{2}}{2G_{i}A\left(x_{i}\right)}+\frac{M_{bi}{}^{2}}{2E_{i}I\left(x_{i}\right)}\right)dx_{i}\right]$$

***N***, ***S*** and ***M_b_*** respectively represent axial force, shear force and bending moment, and are given in Appendix A (A2). According to the second displacement theorem, the displacement in the unit direction is equal to the partial derivative of the overall strain energy against the force acting in this direction, which can be presented as follows:

(9) $$\{\begin{array}{l} u_{6x}=\frac{\partial U}{\partial F_{6x}}=\overset{n}{\underset{i=1}{\sum }}\left(\int_{0}^{l_{i}}\frac{N_{i}}{E_{i}A\left(x_{i}\right)}\frac{\partial N_{i}}{\partial F_{6x}}dx_{i}+\int_{0}^{l_{i}}\frac{\alpha S_{i}}{G_{i}A\left(x_{i}\right)}\frac{\partial S_{i}}{\partial F_{6x}}dx_{i}+\int_{0}^{l_{i}}\frac{M_{bi}}{E_{i}I\left(x_{i}\right)}\frac{\partial M_{bi}}{\partial F_{6x}}dx_{i}\right)\\ u_{6y}=\frac{\partial U}{\partial F_{6y}}=\overset{n}{\underset{i=1}{\sum }}\left(\int_{0}^{l_{i}}\frac{N_{i}}{E_{i}A\left(x_{i}\right)}\frac{\partial N_{i}}{\partial F_{6y}}dx_{i}+\int_{0}^{l_{i}}\frac{\alpha S_{i}}{G_{i}A\left(x_{i}\right)}\frac{\partial S_{i}}{\partial F_{6y}}dx_{i}+\int_{0}^{l_{i}}\frac{M_{bi}}{E_{i}I\left(x_{i}\right)}\frac{\partial M_{bi}}{\partial F_{6y}}dx_{i}\right)\\ \theta_{6z}=\frac{\partial U}{\partial M_{6z}}=\overset{n}{\underset{i=1}{\sum }}\left(\int_{0}^{l_{i}}\frac{M_{bi}}{E_{i}I\left(x_{i}\right)}\frac{\partial M_{bi}}{\partial M_{6z}}dx_{i}\right)=0 \end{array}$$

And the formula above can be simplified as:

(10) $$\{\begin{array}{l} u_{6x}=a_{11}\cdot F_{6x}+a_{12}\cdot F_{6y}+a_{13}\cdot M_{6z}\\ u_{6y}=a_{21}\cdot F_{6x}+a_{22}\cdot F_{6y}+a_{23}\cdot M_{6z}\\ \theta_{6z}=a_{31}\cdot F_{6x}+a_{32}\cdot F_{6y}+a_{33}\cdot M_{6z}=0 \end{array}$$

The coefficient ***a_ij_*** in the formula are given in Appendix A (A3–A5). At the same time, ***F*_6*y*_** is the reaction force from the fiber grating, and ***u*_6*y*_** should be the axial elongation of the fiber grating. According to material mechanics, there are:

(11) $$u_{6y}=b\cdot F_{6y}=-\frac{2F_{6y}\cdot h}{E_{1}\cdot \pi d^{2}}$$

wherein, ***E*_1_**, ***d*** and ***h*** represent the elastic modulus, diameter, and length of the fiber grating, respectively. By combining the above two Formulas (10) and (11), the displacement magnification ratio of the structure can be obtained by Formula (12) and given in Appendix A (A6):

(12) k1=u6yu6x

The structural parameters of the sensor designed in this paper are shown in the Table 1. The structure is made of a copper alloy. ***u*** is the Poisson’s ratio of the structure, and ***E*** is the elastic modulus of the structure. ***L*_1_**, ***L*_3_** and ***L*_5_** is the length of three rigid rods, respectively, and ***α*** is the inclination of the rigid rod in the middle. ***L*_2_** and ***L*_4_** are the lengths of the flexible hinges, which are twice the radius of the hinge for the straight circular flexible hinge selected herein. ***r*** is the radius of the straight circular flexible hinge, and ***t*** is the narrowest width of the flexible hinge. And ***w*** represents the thickness of the flexible hinge. ***E*_1_** is the elastic modulus of FBG, ***d*** and ***h*** represent the diameter and length of FBG respectively.

These parameters were brought into the equations. The Scientific computing software Mathematica was used to solve the simultaneous equations. The final magnification ratio *k*_2_ is 3.21. Meanwhile, the bonding length of FBG is shortened by 3.87 times. In other words, the paste shorten multiple *k*_2_ is 3.87. It can be concluded that the sensitivity of the sensor is 12.42.

## 3. Simulation Analysis of Dynamic Properties of Sensors

### 3.1. Static Performance Simulation

In the previous section, the theoretical model was used to obtain the magnification of the sensor. In this section, the finite element analysis of the structure is carried out by the software of ANSYS to verify the theoretical sensitization multiples obtained in the previous section.

**Figure 5:**
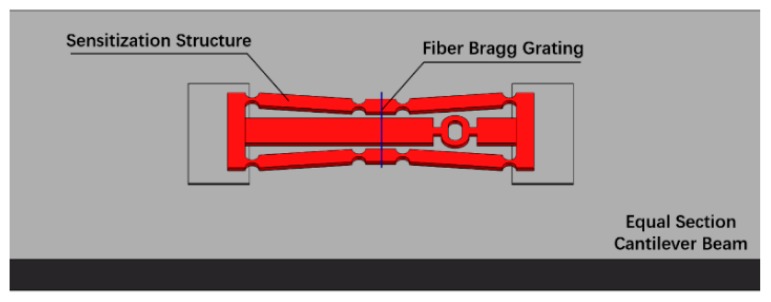
Assembly 3D model.

Considering that the overall rigidity of the sensor is small, optical fiber tension should be taken in account in this simplified simulation model. The sensitizing structure is pasted horizontally on the uniform beam, the fiber grating is then fixed to the sensitizing structure. A three-dimensional model of the sensor was built and the various parts were assembled so that different parts can share the geometric elements at the touch points, as shown in Figure 5. The physical parameters of each part of the main structure are shown in the Table 2.

**Figure 6:**
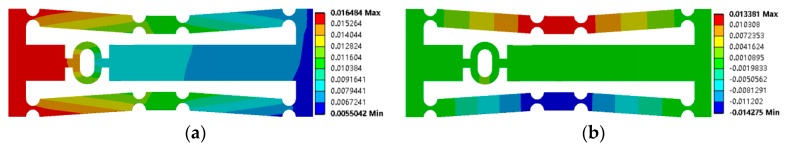
Simulation diagram of sensor. (**a**) Horizontal deformation, (**b**) Vertical deformation.

In the simulation, one end of the beam is fixed, and the horizontal uniform pulling force is applied on the other end. The displacement in the horizontal and vertical direction of the sensor are shown in Figure 6a,b, respectively. These two figures indicate that the deformation of the sensor is uniform and stable. The displacement amplification factor obtained by simulation is 3.01. Therefore, according to Formula (5), the magnification of the sensor is 11.64. The error between theoretical calculation and simulation analysis is within 7%.

### 3.2. Dynamic Performance Simulation

**Figure 7:**
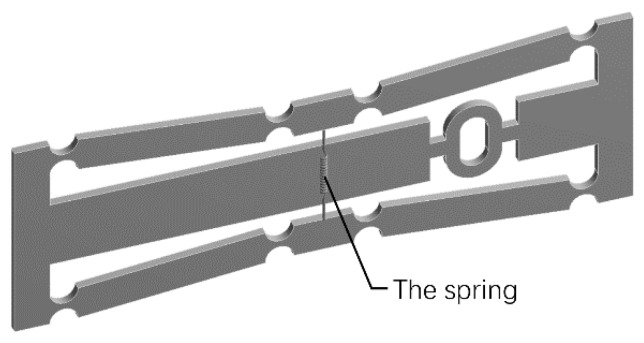
Dynamic simulation model.

Harmonic response analysis is performed to obtain the response of the sensor to dynamic loads. In practice, the pre-stretching of FBG is an important step. The simulation software ANSYS can’t simulate the pre-stretching of the optical fiber entity, so the spring module is used to equivalent the optical fiber in this simulation. The spring is fixed to the sensor by coordinate positioning, as shown in Figure 7. The spring stiffness is set to 157 N/mm, which is calculated according to the elastic modulus, diameter and length of the optical fiber.

**Figure 8:**
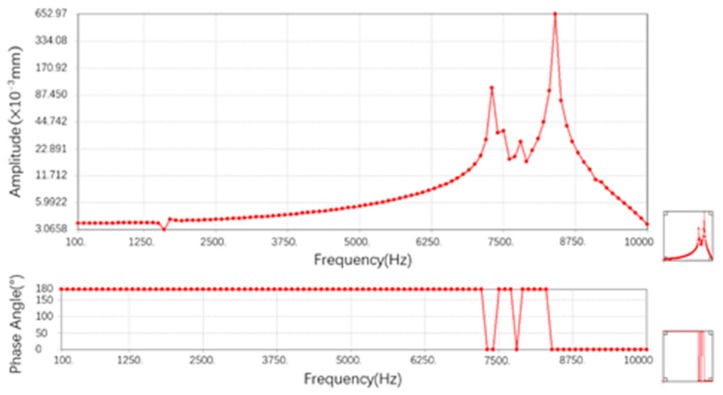
Harmonic response results from 0 Hz to 10 000 Hz.

The sensor is mainly used to measure the axial strain, so the left end of the sensor is fixed and the sinusoidal force of 20 N is applied to the right end. The maximum displacement of Y direction on one side of spring is taken as output. Setting the frequency range to 0–10,000 Hz, a total of 100 equal points, the results of harmonic response analysis are shown in Figure 8.

Above Figure 8 is the amplitude-frequency curve in the frequency range, and below is the phase-frequency curve. It can be obtained that the displacement of the spring end y direction reaches the maximum amplitude at about 7600 Hz and about 8400 Hz, and the phase angle changes by 180°. It can be considered that the natural frequencies of the sensor in the working direction are 7400 Hz and 8600 Hz. At these two frequencies, the substrate resonates in the direction of force. The sensor works well when the force frequency is below 7400 Hz. It can be considered that the sensor basically meets the monitoring requirements of dynamic strain in most mechanical systems.

## 4. Experimental Analysis of Sensing Properties

**Figure 9:**
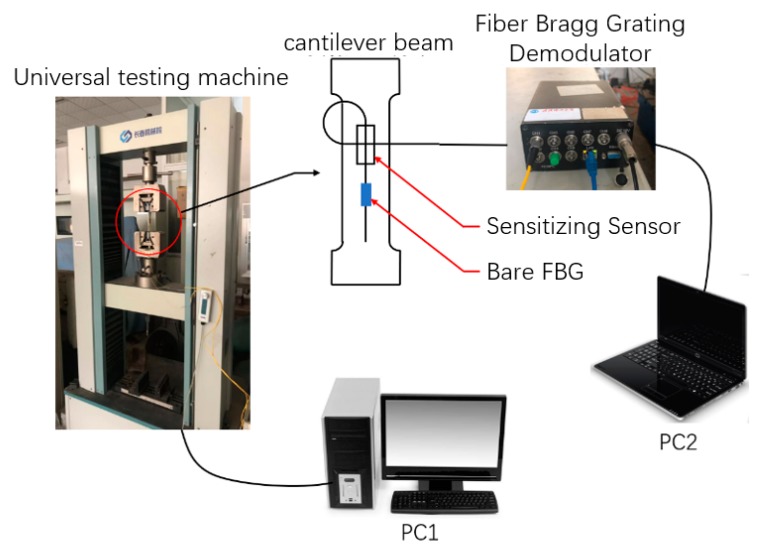
Experimental platform construction.

The strain calibration test was processed on a universal testing machine (Instron 1341). The test platform was built as shown in Figure 9. The two ends of the sensor were spot welded onto the middle of the equal section cantilever beam. The surface bonding FBG was pasted on the side of the sensor to form the control group. The tensile tester first loaded from 0 to 2 kN with a step length of 0.25 kN periodically in three loops. The loading process cycles every 20 s: the loading force increases by 10 s, and then the final load is maintained for 10 s.

**Figure 10:**
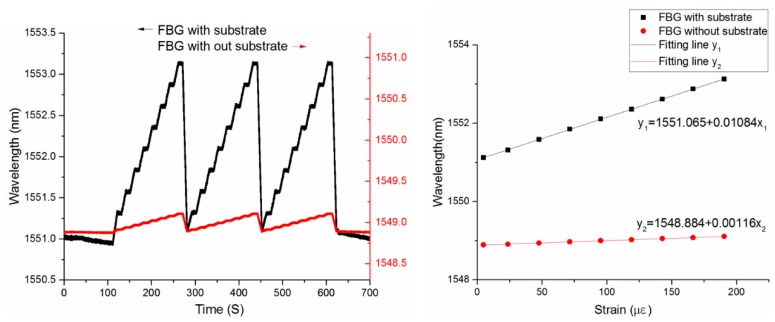
The experiment results. Left: Strain calibration curve; Right: Fitting curve of sensitivity.

The experiment results are shown in Figure 10a. Compared with the bare FBG, the center wavelength drift of the sensor is more evident, which successfully reflects the micro strain on the surface of the cantilever beam. The sensitivity of the sensor with substrate and the bare FBG is fitted in Figure 10b. The results show that the strain sensitivity of the FBG sensor with amplifier substrate can achieve 10.84 pm/με, which is about 10 times to that of the bonding FBG sensor (1.16 pm/με). The sensor sensitization multiple obtained by the static calibration experiment is 9.33. There is a 19.5% error between the experimental results and the theoretical calculation, and a 13.5% error between the experimental results and the simulation. Both the packaging and the installation of the sensor are packaged with the adhesive. The multiple use and the low elastic modulus of the adhesive cause a large loss of strain during the transfer process. As a result, the sensitivities measured by the experiments do not fully meet the theoretical and simulation results.

## 5. Conclusions

This paper proposed and demonstrated a novel strain amplifying sensor for strain measurement with fiber Bragg gratings based on flexure hinge. The results demonstrate that the experimental strain sensitivity of the sensor is 10.84 pm/με, which is about 10 times to bare FBG sensor. It has been found that this FBG strain sensor works well in the temperature up to 65 °C. The new sensor is smaller in size, resistant to electro-magnetic interference and with high-sensitivity, allowing the sensor to be used for accurate dynamic strain monitoring of mechanical structures in operational conditions. Moreover, it also has high stability and repeatability, which ensures the reliability of repeated and long-term monitoring. An array of wavelength-multiplexed FBG sensors installed with this dedicated transducer, therefore, promises to overcome some of the challenges encountered in sensor systems for vibration-based damage identification.

## Figures and Tables

**Table 1:** The structural parameters of the substrate.

Parameter		Parameter	
*U*	0.34	*R* (m)	0.8 × 10^−3^
*E* (GPa)	110 × 10^9^	*W* (m)	0.5 × 10^−3^
*T* (m)	0.4 × 10^−3^	*L*_1_ (m)	5 × 10^−3^
*Θ* (°)	5	*L*_2_ (m)	1.6 × 10^−3^
*E*_1_ (GPa)	70 × 10^9^	*L*_3_ (m)	10.64 × 10^−3^
*D* (m)	125 × 10^−6^	*L*_4_ (m)	1.6 × 10^−3^
*H* (m)	3 × 10^−3^	*L*_5_ (m)	1.7 × 10^−3^

**Table 2:** Material parameters used in simulation.

Component	Young Modulus (GPa)	Poisson Ratio
Substrate	110	0.34
FBG	74	0.33
Beam	200	0.30

## Appendix A

(A1) $$\{\begin{array}{l} C_{11}=\frac{1}{wE}\int_{0}^{l_{i}}\frac{dx_{i}}{t\left(x_{i}\right)}\\ C_{22}=\frac{12}{wE}\int_{0}^{l_{i}}\frac{x_{i}{}^{2}dx_{i}}{t{\left(x_{i}\right)}^{3}}+2\alpha \left(1+\mu \right)C_{11}\\ C_{23}=\frac{12}{wE}\int_{0}^{l_{i}}\frac{x_{i}dx_{i}}{t{\left(x_{i}\right)}^{3}}\\ C_{23}=\frac{12}{wE}\int_{0}^{l_{i}}\frac{dx_{i}}{t{\left(x_{i}\right)}^{3}} \end{array}$$

(A2) $$\{\begin{array}{l} N_{54}=F_{6x}\\ S_{54}=F_{6y}\\ M_{b,54}=M_{6z}+F_{6y}\left(x+l_{5}\right)\\ N_{32}=F_{6x}\\ M_{b,32}=M_{6z}+F_{6y}\left(l_{5}+l_{4}+l_{3}\cdot cos\theta +x\right)-F_{6x}\cdot l_{3}\cdot sin\theta \end{array}$$

(A3) $$\{\begin{array}{l} a_{11}=l_{3}{}^{2}sin\theta^{2}\cdot C_{33}+2C_{11}\\ a_{12}=-l_{3}sin\theta \left[\left(\left(l_{5}+l_{4}+l_{3}con\theta \right)\cdot C_{33}+C_{23}\right)\right]\\ a_{13}=-l_{3}sin\theta \cdot C_{33} \end{array}$$

(A4) $$\{\begin{array}{l} a_{21}=-l_{3}\cdot sin\theta \cdot \left[\left(l_{5}+l_{4}+l_{3}con\theta \right)\cdot C_{33}+C_{23}\right]\\ a_{22}=2C_{22}+2\left(2l_{5}+l_{4}+l_{3}cos\theta \right)\cdot C_{23}+{\left(l_{5}+l_{4}+l_{3}con\theta \right)}^{2}\cdot C_{33}+l_{5}{}^{2}\cdot C_{33}\\ a_{23}=2C_{23}+2\left(2l_{5}+l_{4}+l_{3}cos\theta \right)\cdot C_{33} \end{array}$$

(A5) $$\{\begin{array}{l} a_{31}=-l_{3}\cdot sin\theta \cdot C_{33}\\ a_{32}=2C_{23}+2\left(2l_{5}+l_{4}+l_{3}cos\theta \right)C_{33}\\ a_{33}=2C_{33} \end{array}$$

(A6) $$k_{2}=\frac{ba_{33}\left(a_{23}a_{32}-a_{22}a_{33}\right)}{a_{33}\left(a_{12}a_{23}a_{32}+a_{11}a_{22}a_{33}-a_{11}a_{23}a_{32}-a_{12}a_{22}a_{33}+a_{13}a_{32}b-a_{11}a_{33}b\right)}$$
